# Controlling Seizure-Like Events by Perturbing Ion Concentration Dynamics with Periodic Stimulation

**DOI:** 10.1371/journal.pone.0073820

**Published:** 2013-09-16

**Authors:** Jeremy A. Owen, Ernest Barreto, John R. Cressman

**Affiliations:** 1 King’s College, University of Cambridge, Cambridge, United Kingdom; 2 School of Physics, Astronomy, and Computational Sciences and The Krasnow Institute for Advanced Study, George Mason University, Fairfax, Virginia, United States of America; SUNY Downstate MC, United States of America

## Abstract

We investigate the effects of adding periodic stimulation to a generic, conductance-based neuron model that includes ion concentration dynamics of sodium and potassium. Under conditions of high extracellular potassium, the model exhibits repeating, spontaneous, seizure-like bursting events associated with slow modulation of the ion concentrations local to the neuron. We show that for a range of parameter values, depolarizing and hyperpolarizing periodic stimulation pulses (including frequencies lower than 4 Hz) can stop the spontaneous bursting by interacting with the ion concentration dynamics. Stimulation can also control the magnitude of evoked responses to modeled physiological inputs. We develop an understanding of the nonlinear dynamics of this system by a timescale separation procedure that identifies effective nullclines in the ion concentration parameter space. Our results suggest that the manipulation of ion concentration dynamics via external or endogenous stimulation may play an important role in neuronal excitability, seizure dynamics, and control.

## Introduction

Seizure control by periodic electrical stimulation is a promising avenue for the treatment of refractory epilepsies [1], [2]. Experimental studies have investigated a variety of electrical stimulation protocols, observing seizure suppression both in humans [3]–[6] and in non-human animal or *in vitro* models of epilepsy [7], [8]. However, the mechanisms that underlie this type of control are not well understood [9], [10].

Here we propose a basic mechanism for seizure suppression that is based on the perturbation of ion concentration dynamics by electrical stimulation. The significance of ionic imbalance in seizures is well established, and the various roles it plays in cellular and network excitability are currently of significant interest [11]. Electric currents–whether they promote or suppress neuronal activity–directly impact the ion concentrations within and surrounding the affected cells, and therefore influence the electrochemical drive of ions across the neuronal membrane.

The mathematical model we analyze is of a single neuron, augmented to include dynamic intracellular sodium and (local) extracellular potassium concentrations. Our model is simple and generic, and excludes a number of the biological mechanisms known to be at work. Although this simplicity somewhat weakens the quantitative predictions of the model, we believe it leaves the qualitative results intact, and indeed makes them more general. In particular, our main qualitative results do not require finely-tuned model parameters. Instead, they arise merely from gross features of the ion dynamics.

We previously studied the role of ion concentration dynamics in a similar model and identified bifurcations to stable limit cycles which correspond to very slow (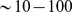 s) modulation of the ion concentrations. These modulations drive the neuron into, and out of, the spiking state, and thus give rise to bursting/seizing behavior. This behavior can be attained by a choice of parameters similar to those seen in experiments (e.g., brain slice preparations in elevated potassium [12], [13]). Here we show how stimulation can interact with this limit cycle and, under a wide range of parameters, effectively stop it.

By framing stimulation in the context of the ionic dynamics of a single neuron, our results may shed light upon the mechanism of action of direct brain stimulation as a treatment for epilepsy. Our model is also useful for investigating threshold behavior for seizure generation that results from physiological inputs.

## Methods

Our model is a modification of the Hodgkin-Huxley neuron to include dynamic intra- and extracellular ion concentrations. This model has been previously described and analyzed in [14] and in [15]. It has also been extended (by others) to model and explain phenomena observed in rat EEG traces under conditions of oxygen and glucose deprivation [16].

The model consists of equations that describe spiking behavior,
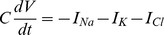


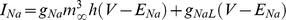


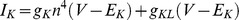


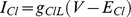
plus two differential equations that model the time evolution of the local extracellular potassium (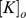) and the intracellular sodium (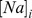) concentrations:



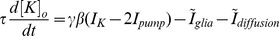


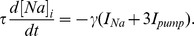



In these equations, 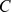 is the membrane capacitance, 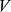 is the membrane potential, and the 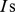 represent membrane ion current densities. The 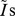 are molar currents (millimolars per second). The parameter 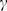 is a unit conversion factor, and 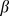 is the ratio of intracellular to extracellular volume. These geometric parameters are derived based on the assumption of a spherical cell [14], [15], and we neglect the electrogenic contribution of the pump to the voltage equation. With the dimensionless parameter 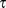 set to 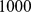, the units of time are set to milliseconds.

Two assumptions, introduced in [14], simplify the model and permit analysis. We adopt these here as well:
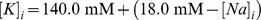


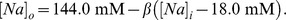



Throughout the remainder of this report, we refer to 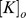 as “potassium” and 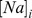 as “sodium” where the context is clear. We do not discuss 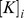 and 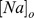 explicitly.

The maximum membrane conductances for sodium, potassium, and chloride are 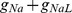, 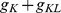, and 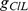, respectively, where the subscript L indicates a leak conductance. The reader is referred to [15] for the equations describing the time evolution of the gating variables 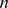 and 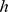; see also Table 1. 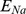 and 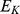 are the equilibrium potentials for sodium and potassium, respectively. These depend on the ion concentrations outside and within the neuron:
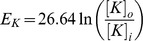


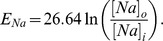



**Table 1:** Variables and parameters.

Symbol	Unit/Default Value	Description
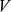	mV	Membrane potential
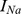	*μ* A/cm^2^	Sodium current
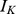	*μ* A/cm^2^	Potassium current
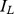	*μ* A/cm^2^	Leak current
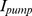	*μ* A/cm^2^	Pump current
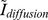	mM/s	Potassium diffusion to the nearby reservoir
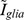	mM/s	Glial uptake
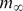	1	Activating sodium gate
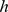	1	Inactivating sodium gate
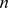	1	Activating potassium gate
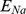	mV	Reversal potential of sodium current
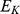	mV	Reversal potential of potassium current
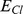	mV	Reversal potential of chloride current
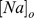	mM	Extracellular sodium concentration
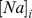	mM	Intracellular sodium concentration
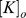	mM	Extracellular potassium concentration
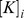	mM	Intracellular potassium concentration
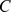	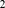 1 *µ*F/cm	Membrane capacitance
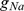	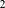 100 mS/m	Conductance of persistent sodium current
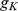	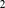 40 mS/m	Conductance of potassium current
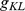	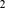 0.05 mS/m	Conductance of potassium leak current
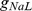	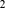 0.0175 mS/m	Conductance of sodium leak current
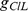	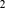 0.05 mS/m	Conductance of chloride leak current
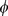	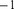 3 s	Time constant of gating variables
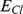	−81.94 mV	Reversal potential of chloride current
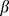	7.0	Ratio of intracellular to extracellular volume of the cell
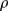	1.25 mM/s	Pump strength
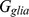	66 mM/s	Strength of glial uptake
	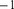 1.33 s	Diffusion constant
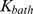	4.0 mM	Potassium concentration of extracellular reservoir
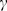	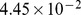	Conversion factor

Chloride dynamics are not modeled here, so we set 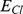 to the constant value 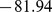 mV.


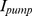
 and the molar currents 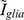 and 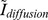 depend on the ion concentrations as follows:
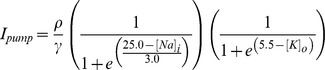


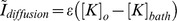


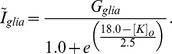



The pump strength is 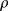 and the strength of glial buffering is 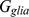. The rate of diffusion is controlled by the parameter 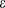. 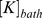 corresponds to the concentration of potassium in the reservoir surrounding the model neuron (i.e., the bathing solution in the case of a slice preparation, or the vasculature *in vivo*).

Together, 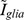 and 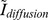 allow for the flow of potassium both to and from the extracellular space.

### Adding Stimulation

To model electrical stimulation, a term is simply added to the voltage equation, in accordance with the sign convention in [17]:
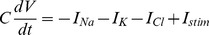
where 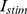 is a series of square pulses:



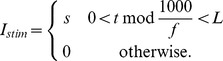



Here, 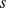 is the strength in 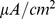, 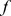 is the frequency of the stimulation in hertz, and 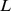 is the length of each pulse in milliseconds. Throughout the rest of this paper, stimulation protocols will be identified by the three numbers 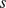, 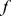, and 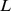. Where not specified, stimulation was done using 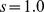 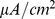 and 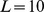 ms. These values were chosen so that each stimulation pulse elicits an action potential in the elevated 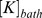 condition in which we seek to apply control. They are not the only values of 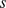 and 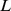 that elicit action potentials (and therefore other parameter choices give rise to results qualitatively similar to those we report).

Numerical simulations of the model were run in Wolfram Mathematica and C.

### Separation of Timescales

Throughout the paper we make reference to potassium and sodium “nullclines” that arise for a given choice of model parameters. Formally, these are the curves defined by 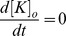 and 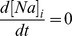, which we plot in the ion concentration phase space. At first glance it seems that these curves must also depend on the values of the other dynamical variables, 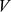, 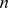, and 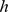
–and indeed the differential equations for the ion concentrations do, via the currents 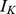 and 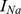.

Fortunately, the behavior of the model can be divided sharply by timescale. There is fast (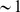 ms) spiking behavior governed by 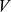, 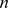, and 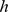, and slow (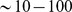 s) 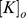 and 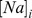 dynamics. Whenever we mention the sodium or potassium “nullclines”, we refer to curves across which the *time-average* of 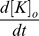 or 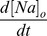 changes sign. These curves reflect the slow ion concentration dynamics much as true nullclines would, e.g., equilibria are at intersections of these curves, and trajectories cross the curves approximately perpendicular to one axis or the other.

The time-averaged nullclines are generated point by point and then connected to form the curves shown in the figures. For fixed 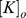, a bisection algorithm is used to approximate the zero of 
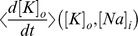
 or 
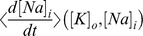
, where the angle brackets denote time-averaging. Note that this method assumes there is only one point on each such “nullcline” for each 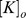 value. It is possible to check that this holds by looking at the ion concentration dynamics and noting that each concentration variable changes direction only once as 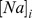 is increased–that is, the “nullclines” appear to be graphs (in the mathematical sense) of *functions* from 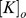 to 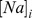.

The separation of timescales described above is similar to the model reduction performed in [14]. In that work, the timescale separation was accomplished by manually fitting functions to approximate the time-averaged current surfaces 
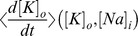
 and 
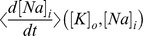
–a method which has difficulty achieving arbitrary accuracy, unlike the method used here (for more accuracy, simply run more iterations of the bisection algorithm).

### Thresholds of the Model

We will describe a number of phenomena relating to our model neuron’s response to inputs and changes in exogenous parameters. Many of our explanations for these phenomena will appeal to the notion of thresholds of the model. Because this term can be interpreted in several ways, we clarify here what we mean.

If we choose *fixed* values of the ion concentrations at various points in the 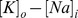 ion concentration phase space, it becomes clear that there is a large region where our model neuron exhibits tonic spiking if placed there. The boundaries of this region were described in [15]. Of relevance here is the boundary defined by the codimension-one SNIC (saddle-node on an invariant circle) bifurcation curve, which separates the spiking region from a region in which the neuron is attracted to a resting state equilibrium.

Consider now our full dynamic model, in which the ion concentrations are dynamic variables. For putatively normal values of the 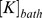 parameter, the full model is attracted to the resting equilibrium. As 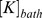 is increased beyond a critical value, periodic bursting suddenly appears with the creation of a limit cycle [15]. On this limit cycle, the ion concentration variables oscillate in such a way as to repeatedly cross the SNIC bifurcation boundary described above. That is, the neuron repeatedly transitions from the resting region to the spiking region and back again, and bursting behavior is seen. Thus, the SNIC bifurcation curve can be thought of as a threshold for spiking. On the other hand, the critical value of the parameter 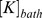 can also be thought of as a threshold for the onset of periodic bursting.

Later in this paper we will consider transient bursts, in which the full model, under conditions in which it is attracted to the resting equilibrium, is perturbed so as to kick the neuron across the SNIC boundary and into the spiking region. As the neuron then relaxes back to a resting equilibrium, a transient burst of spikes is observed. We will show below that the potassium nullcline described above serves as another kind of threshold, one which separates qualitatively different versions of these transient bursts.

## Results

Previous work [14], [15] has demonstrated that, in the absence of stimulation, the model described above undergoes bifurcations to various spontaneous bursting states as parameters such as 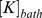 are altered from their default “normal” values (which we take to be 4 mM for 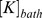). For example, a transition from resting to periodic bursting occurs at 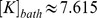 mM.

**Figure 1:**
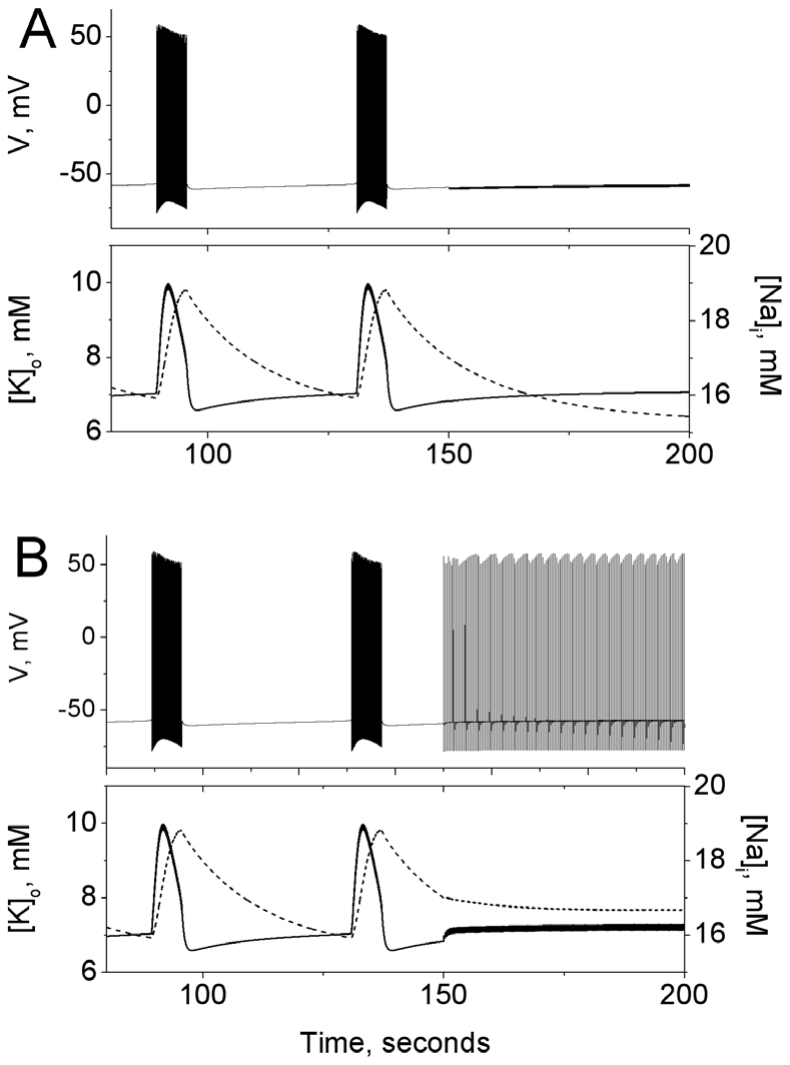
Time traces showing the cessation of bursting oscillations by periodic stimulation. In both panels, the top plots show the transmembrane voltage, and the double Y plots below show the evolution of 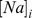 (dashed line) and 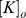 (solid line). With 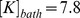 mM, the unperturbed model exhibits periodic bursting. At 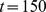 seconds, (**A**) inhibitory (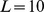 ms, 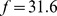 Hz, 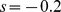 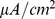) or (**B**) excitatory (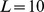 ms, 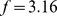 Hz, 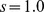 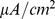) periodic stimulation is turned on. In both cases the large oscillations in the ion concentrations cease, but in the excitatory case, the neuron spikes at the frequency of stimulation.

The resulting behavior is shown in Figure 1 for 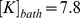 mM. For 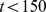 seconds, the model exhibits spontaneous, periodic bursting. Two bursts are shown. In the voltage time traces (top of each panel), the bursts appear as clusters of spikes, and in the ion concentration time traces (the double Y plots at bottom of each panel), they appear as long, slow modulations. Note the large values of 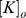 during the bursts, and the slow decay of 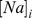 during the quiet phases.

The main result of this work–the cessation of bursting in this generic neuron model due to periodic stimulation–is also illustrated in Figure 1. Spontaneous bursting in the model is halted by the addition of periodic current injections starting at 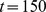 seconds. In (A), the stimulation is inhibitory (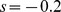 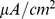, 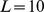 ms, and 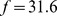 Hz), and spikes in voltage are prevented from occurring. In (B), the stimulation is excitatory (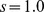 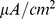, 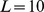 ms, and 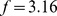 Hz), and the model continues spiking, but only at the stimulation frequency–which can be made much lower than the intrinsic firing rates seen in the model. In both cases, the ion concentrations approach relatively constant values under periodic stimulation.

### Controlling Spontaneous Behavior

In this section we consider the interaction between periodic stimulation and the spontaneous behavior of the model, particularly the stable limit cycle that corresponds to bursting, which appears at 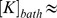 7.615 mM. We explore how stimulation can interrupt the limit cycle, effectively terminating the bursting.

**Figure 2:**
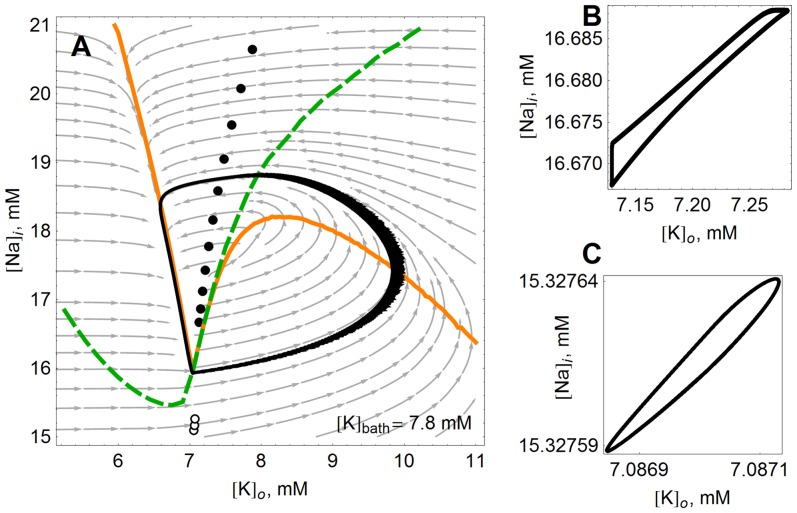
Model dynamics visualized in the 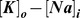 phase plane. In these panels, the unperturbed behavior of the model is compared to the controlled behavior at 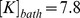 mM. (**A**) The model’s unperturbed behavior is the large black limit cycle. 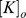 and 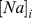 nullclines are drawn in orange and (dashed) green, respectively. With excitatory periodic stimulation (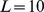 ms, 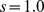 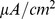), the neuron moves to the “pseudoequilibria” denoted by solid circles. The pseudoequilibria move higher in 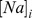 for increasing frequency, ranging from 3.16 Hz to 31.6 Hz in even logarithmic increments. Open circles denote pseudoequilibria for inhibitory stimulation (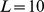 ms, 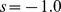 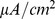), moving down in 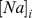 as frequency increases from 15.8 Hz to 25.1 Hz. The pseudoequilibria denoted by circles in panel (A) are in fact very small limit cycles, shown in panels (**B**) and (**C**) in the excitatory and inhibitory case, respectively. Note the scales in (B) and (C) compared to (A).

To illustrate these dynamics, it is useful to consider the 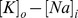 phase plane, on which the projection of a bursting limit cycle appears as a “loop” [15]. See Figure 2. On this diagram the black loop shows the trajectory of the ion concentrations with no stimulation–this is another representation of the data in the bottom panels of Figure 1 for 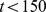 seconds. The model follows the loop in the counter-clockwise direction. During the quiet phases between bursts, the trajectory of the ion concentrations follows the left edge of the black loop as 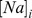 slowly decays. During the spiking phase, the arc to the right is quickly traversed. The apparent thickness in the right part of the arc is due to the individual spikes that make up the burst. The model spikes spontaneously in a large region of the ion concentration phase space.

When periodic stimulation is applied, the ion concentrations approach very small loops which are difficult to distinguish from equilibria on the scale of the unperturbed limit cycle. We refer to these as “pseudoequilibria”. Examples are shown in Figure 2B (excitatory stimulus) and 2C (inhibitory stimulus) (note the scale). The locations of these small loop pseudoequilibria are indicated in Figure 2A for various values of the stimulus frequency, with filled circles denoting points reached due to excitatory stimulation, and open circles denoting the same in the inhibitory case. For excitatory stimulation of increasing frequency, the pseudoequilibria occur in locations increasingly removed from the 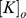 nullcline (orange), and higher in 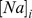. For inhibitory stimulation, faster stimulation sends the model neuron to lower 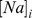 values.

Thus, periodic stimulation achieves control by creating new, much smaller limit cycles in the sodium and potassium concentrations that effectively freeze the large scale dynamics–stopping the model neuron from bursting. These small limit cycles appear because each stimulation pulse forces the ion concentrations to values that then quickly relax back under the intrinsic dynamics in between stimulation pulses. This effect is best understood in terms of the flow field (gray) in the background of Figure 2A. Excitatory stimulation results in the increase of both 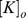 and 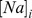; stabilization can therefore occur if the stimulation kicks the system to a region where the intrinsic sodium and potassium flow is negative. Releasing the system from control here leads to a return to the limit cycle (taking longer for higher frequencies) by way of the potassium nullcline. In contrast to excitatory stimulation, inhibition decreases 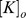 and 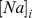, and therefore inhibitory stimulation can only be balanced in a region where the intrinsic flows are positive (e.g. sodium into the cell, potassium out). Abruptly releasing the system from control in this region of phase space leads to a seizure-like response even larger than the intrinsic bursts.

In our model, seizure control via electrical stimulation is robust to variations in the stimulation rate. This is because during the recovery of the ion concentrations after a burst of spikes, the rate of descent in 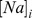 slows smoothly as the neuron approaches the cusp of the potassium nullcline. If this slowing did not occur, periodic excitatory stimulation would need to be finely tuned to freeze the dynamics.

#### Robustness of control

For a given choice of 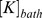, what range of stimulation frequencies can stop bursting? Conversely, for a given choice of stimulation frequency, how high can 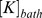 be pushed before the model bursts in spite of the stimulation?

**Figure 3:**
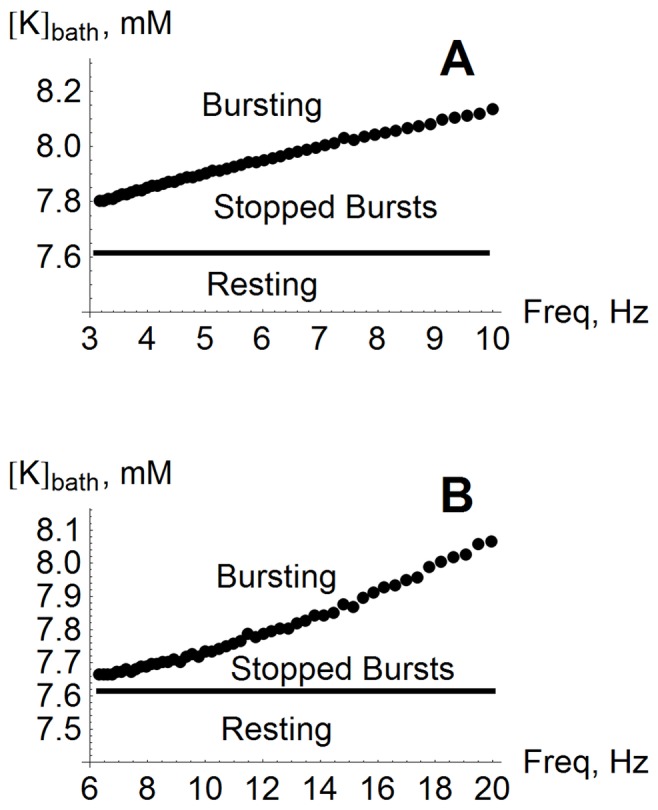
Control with higher simulation frequency is more robust. With no stimulation, spontaneous periodic bursting appears at 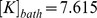 mM. This behavior can be controlled with periodic stimulation. The upper boundary of this controllable region shifts higher in 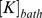 for higher stimulus frequency with both (**A**) excitatory stimulation (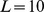 ms, 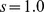 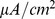), and (**B**) inhibitory stimulation (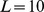 ms, 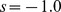 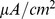).

These natural questions can be answered by mapping out the major behavioral transitions in the frequency-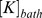 parameter space. The results of this are shown in Figure 3. The bursting threshold value for 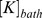 is plotted versus the frequency of (A) excitatory or (B) inhibitory stimulation. In both charts, the solid black horizontal line at 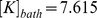 mM is the bursting threshold with no stimulation. Above this is a region in which our control protocol is effective. The main finding is that the upper boundary of this control region shifts to higher values of 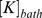 for higher stimulation frequencies, thus expanding the parameter range in which bursting can be stopped. This implies that control using higher frequencies is more robust to changes in the potassium bath concentration.

Excitatory stimulation that fails to stop bursts can nevertheless reduce their amplitude. When the model neuron is subjected to excitatory stimulation, the recovery of 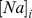 after a burst occurs at elevated 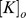. As a result, the next burst is elicited prematurely–before 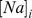 has recovered to normal pre-burst levels–and is therefore stunted in size. This is because, as can be seen in Figure 2, the dynamics are such that the ionic flows reverse direction at higher 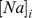 when 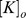 is elevated (e.g. the nullclines slope upwards in the relevant region of the phase plane).

When 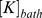 is very close to the 7.615 mM threshold but just below it, periodic stimulation sometimes causes transient bursts to occur before the model settles down to a controlled pseudoequilibrium. We examine the behavior of such transient bursts in the next section.

#### Transient behavior

As long as 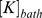 remains below 5.955 mM (approximately), and the system starts at its equilibrium point, stimulation pulses with 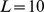 ms and 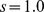 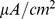 do not elicit an action potential, and therefore have only a tiny effect on the ion concentrations. That is, depolarizing stimulation of this amplitude can be applied to the model with 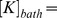 5.955 mM with virtually no consequence.

**Figure 4:**
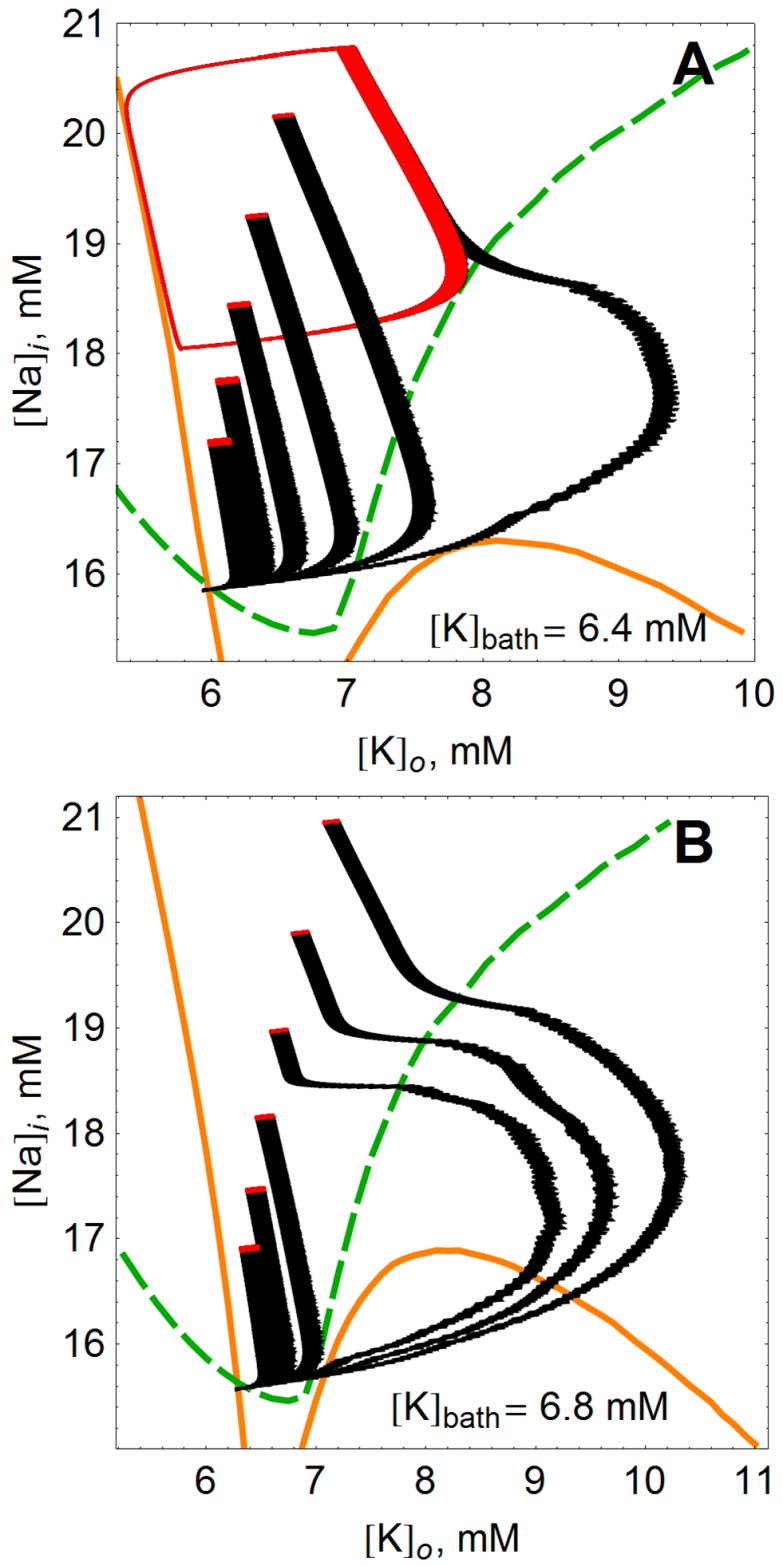
Transients from equilibrium to new steady states due to periodic stimulation at subthreshold 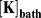. In both panels, trajectories shift from left to right as the frequency of excitatory stimulation (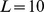 ms, 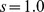 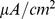) increases from 3.16 Hz to 31.6 Hz, in even logarithmic increments of 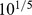. 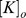 and 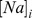 nullclines are drawn in orange and (dashed) green, respectively. 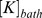 is either (**A**) 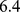 mM or (**B**) 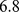 mM.

As 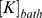 rises, however, this stimulation begins to have an effect, and the system is driven to an increasingly displaced pseudoequilibrium. In Figure 4 we show transients elicited by excitatory periodic stimulation at subthreshold values of 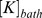, either 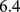 mM or 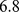 mM in panels (A) and (B), respectively. The frequencies used in both panels are consistent with the stimulation rates required to stop bursts as reported above and range from 3.16 Hz to 31.6 Hz. As expected following the results in Figure 2 (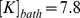 mM, above the bursting threshold), faster stimulation eventually leads to pseudoequilibria higher in 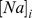. The pseudoequilibria attained with the slowest stimulation (3.16 Hz) are reached after waiting (at most) just slightly longer than one minute. Note also that the locations of the pseudoequilibria do not vary drastically with 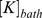.

What is revealed here is that the faster stimulation protocols (the trajectories shown further on the right in each panel) send the model neuron on large excursions towards the right in the 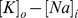 phase space before settling down. This effect is more pronounced in panel (B), where 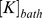 is closer to the onset-of-periodic-bursting threshold. Note that two of the stimulation frequencies that cause large “seizure-like” transients at 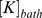 = 6.8 mM do not do so at 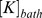 = 6.4 mM. Note also that the displayed nullclines reflect only intrinsic dynamics–stimulation is applied “on top” of these dynamics and can force the trajectories to cross nullclines at the wrong angle (i.e., not perpendicular to either axis), and to settle at points where nullclines do not intersect.

These observations suggest that by temporarily lowering 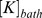 at the beginning of the stimulation, one can avoid the seizure-like excursion through the high 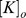 regions of the phase plane. The same effect can be achieved by gradually ramping up the frequency. For instance, at fixed 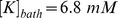, stimulating at 8 Hz for one minute and then increasing the frequency to 12.5 Hz does not elicit a large transient (not shown), whereas stimulating immediately at 12.5 Hz (with the model starting at the equilibrium point) does.

In the low 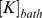 case, panel (A), the fastest stimulation trajectory shown sends the model to a large (roughly rectangular) limit cycle–a stimulus-induced loop. The cycle occurs because the model reaches a region in the phase space where the stimulation pulse cannot initiate a spike in voltage (and therefore has a tiny effect on the ion concentrations). This causes the trajectory to descend along the potassium nullcline (under intrinsic dynamics) until the concentrations are such that the stimulation can elicit spikes again, at which point the trajectory is pushed in the direction of increasing 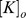 and the cycle repeats. Note that the firing rate along the spiking portion of this limit cycle is the same as the stimulation frequency.

The numerical experiments we conducted to generate Figure 4 were devoted to understanding how our control mechanism might interact with transient changes in 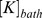 that sometimes occur pathophysiologically in conditions such as epilepsy [18]. We found that as long as 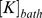 and/or the stimulation frequency are changed slowly, on the order of seconds, large transients are not evoked by the control stimulation.

### Controlling Evoked Behavior

In this section, we consider our model’s response to inputs that might be supplied endogenously at synapses. In particular, we focus on how these inputs can elicit different types of transients, including seizure-like discharges, and how periodic stimulation at the slow controlling frequencies we report above can have a mitigating effect.

#### Large potassium efflux

**Figure 5:**
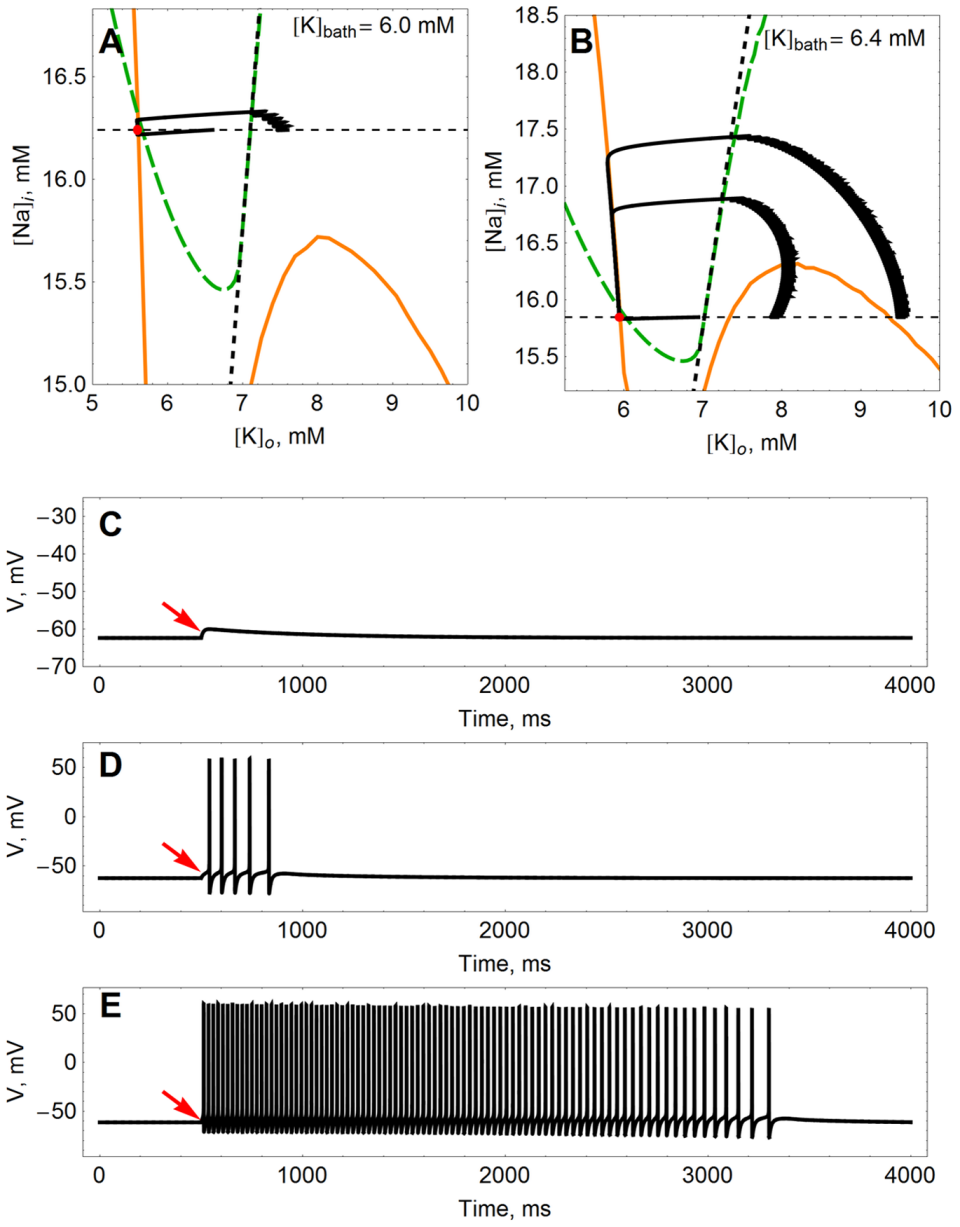
Response of the model to an instantaneous increase in 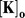
–the K nullcline as a bursting threshold. The position of the nullclines changes with 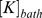, and so too the response of the model to an instantaneous increase in 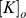. In the phase planes (**A**) and (**B**), the equilibrium point is denoted by a red dot. From this equilibrium, we perturb the model by sliding out along the horizontal dashed line. The black curves show the trajectories of the model recovering from stimuli of different sizes. Potassium (orange) and sodium (dashed green) nullclines are drawn in each case. Also shown is the SNIC curve that marks the onset of spontaneous spiking (short-dashed line running approximately vertically). (**C**) shows a voltage time trace of the small response from (A), (**D**) shows the larger of the transients shown in (A), and (**E**) shows the largest transient in (B). The perturbation from equilibrium occurs at the arrows (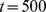 ms).

Figure 5 illustrates how the response of the model to a large, instantaneous potassium ion efflux depends on the location of the nullclines. Both panels show the phase plane for subthreshold values of 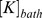 (i.e., no spontaneous bursting). The model is first allowed to settle down under the intrinsic dynamics, which send the ion concentrations to the stable equilibrium at the intersection of the potassium (orange) and sodium (dashed green) nullclines (marked by a red dot). Then we examine the effects of an instantaneous increase in the extracellular potassium concentration, i.e., abruptly shifting rightward along the horizontal dotted line.

In panel (A), 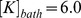 mM and no large response is seen. In particular, the positioning of the nullclines is such that it is not possible to cross the potassium nullcline by moving rightward from the equilibrium point. Following a small shift in 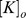, the neuron simply relaxes back to the resting equilibrium, as shown in panel (C), which shows the corresponding voltage time trace. A more significant response is elicited by crossing the SNIC curve (the boundary for the onset of spiking, shown here as a short-dashed, approximately vertical line), which is roughly coincident with a portion of the sodium nullcline. Still, only a few spikes occur; see panel (D).

This stands in stark contrast to the behavior shown in panel (B), where 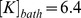 mM and a sufficiently large abrupt increase in 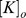 from equilibrium causes the model to cross the potassium nullcline (orange). This is because the increased value of 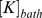 has raised the “knee” of the potassium nullcline, both in absolute terms (e.g. with respect to 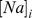) and relative to the location of the equilibrium (where the nullclines cross). The response here is more severe, consisting of a prolonged burst, as illustrated in panel (E).

The purpose of exploring the response to a discontinuous shift in 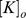 is to establish that the potassium nullcline is effectively a threshold for the occurrence of prolonged seizure-like bursts arising from transient perturbations of the model. The variety of ways the ion concentrations can recover from such shocks is the primary mechanism that controls the length and nature of the transient response. If large inputs move the ion concentrations to a point in the phase space where the sodium and potassium flows are negative (out of and into the cell, respectively), the neuron will recover relatively quickly and will not spike spontaneously. On the other hand, if an input sends the concentrations across one or both nullclines into regions where the ionic flows reverse, the response is significantly more pronounced.

#### Fast periodic stimulation

We now examine the effects of more realistic excitatory inputs. In particular, we use a transient tetanic stimulation consisting of a 50 Hz spike train of various durations, with 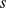 = 0.75 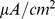 and 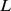 = 10 ms. This is meant to simulate synaptic barrages which can cause significant increases in the local extracellular potassium concentration. In contrast to the discontinuous shifts in 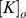 used in the previous section, this tetanic stimulation results in a curved displacement towards higher values of both 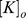 and 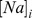 in the ion concentration space, depending on duration.

**Figure 6:**
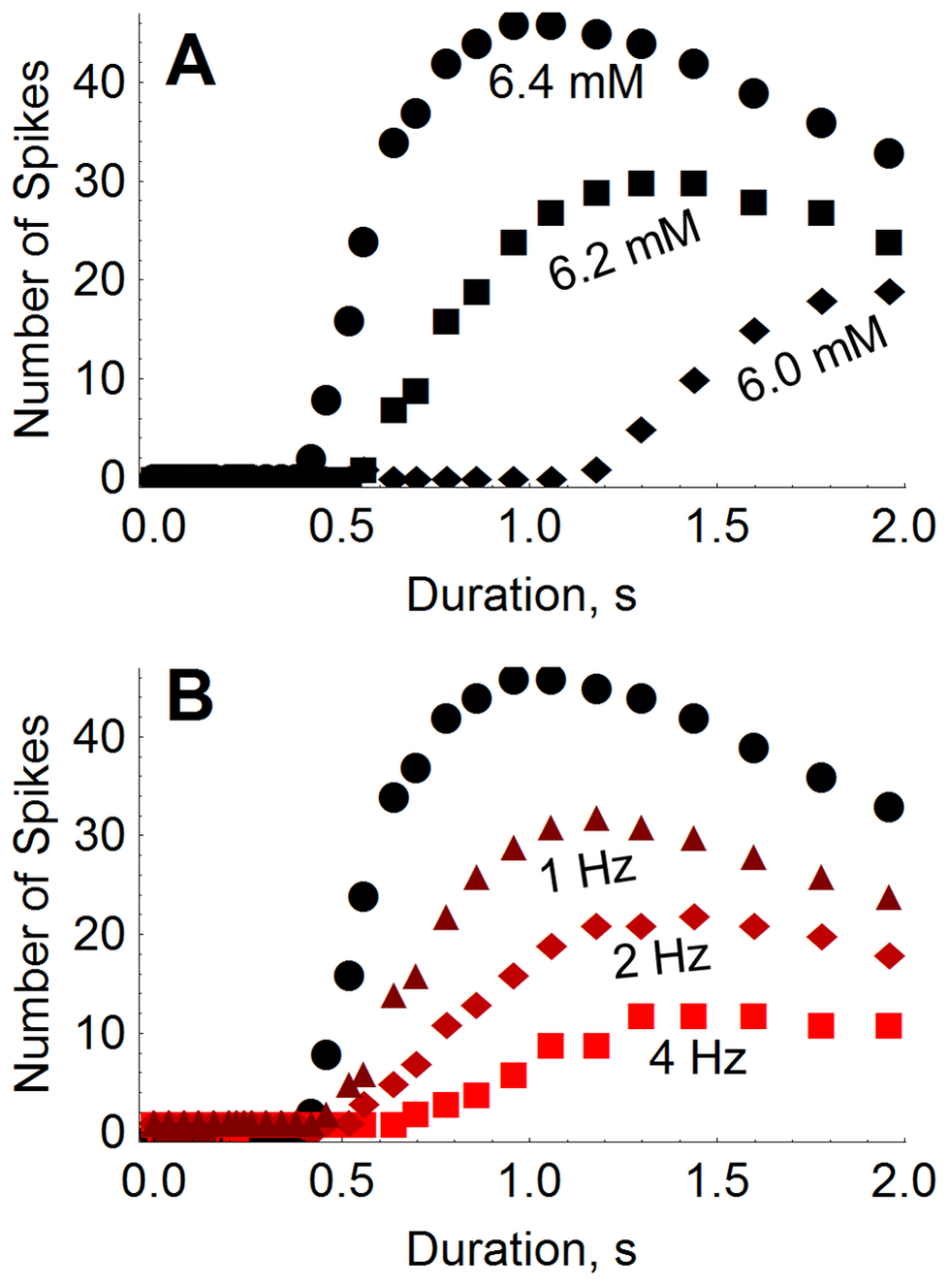
Number of spikes elicited by tetanic stimulation under various conditions. The number of spikes that occur spontaneously after a 50(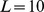 ms, 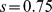 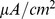) depends on stimulus duration. In panel (**A**), this dependence is shown for 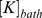 = 6.4 mM, 6.2 mM, and 6.0 mM. If the spike train occurs concurrently with a slow background periodic stimulation (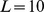 ms, 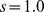 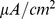), the response is diminished according to the frequency of the slow stimulation–shown in (**B**) for 1 Hz, 2 Hz, and 4 Hz. In (B), 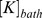 is fixed at 6.4 mM throughout, and the response with no background stimulation is shown in black for comparison.

We plot in Figure 6A the number of spikes elicited after the tetanus versus the tetanus duration, for different values of 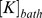. As the tetanus duration is increased, there is essentially no response until a critical duration is reached. This is because the extracellular potassium must accumulate sufficiently so as to cross the spiking threshold (SNIC curve) as in Figure 5. Note that this critical duration is significantly longer for 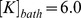 mM due to the fact that the resting equilibrium is farther from the spiking boundary.

The most obvious result is that the model neuron is generally more excitable for larger values of 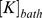
–for the same tetanus duration, higher 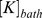 means more elicited spikes. As before, the post-stimulus response depends strongly on the placement of the potassium nullcline. For 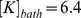, the potassium nullcline is located such that the tetanus can drive the neuron across it, thus leading to a prolonged transient response (see Figure 5). For 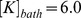, this is not the case, and the increase in the number of elicited spikes is more gradual.

For 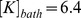 and 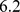 mM, the number of elicited spikes first increases, and then decreases for longer tetanus durations. This is due to the accumulation of intracellular sodium during the tetanic stimulation. As described above, the tetanus quickly drives the neuron along a curved path towards higher 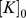 and 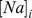. For sufficiently long tetanus durations, the neuron is driven to positions above (not within) the knee, where, once the tetanus ends, the intrinsic dynamics favors a very rapid decrease in 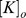 and hence a fast termination of the burst. Thus, the number of elicited spikes decreases.

Next we show that a slow background periodic stimulation can modulate the response to the fast tetanic input described above. We repeated the numerical experiment in Figure 6A for 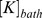 = 6.4 mM in the presence of an ongoing background stimulation at 1, 2, and 4 Hz (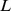 = 10 ms, 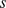 = 1.0 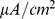). The background stimulation was in effect before, during, and after the tetanus was applied. Results are shown in Figure 6B. A decrease in the number of elicited spikes is seen as the background stimulation frequency increases. This can be understood by recalling the position of the controlled pseudoequilibria shown in Figures 2A and 4A. With increasing background stimulation frequency, the pseudoequilibria occur at higher sodium concentrations, are increasingly farther from the potassium nullcline, and eventually move higher than its knee. The subsequent response to the tetanic stimulation is therefore diminished.

#### Response skipping

**Figure 7:**
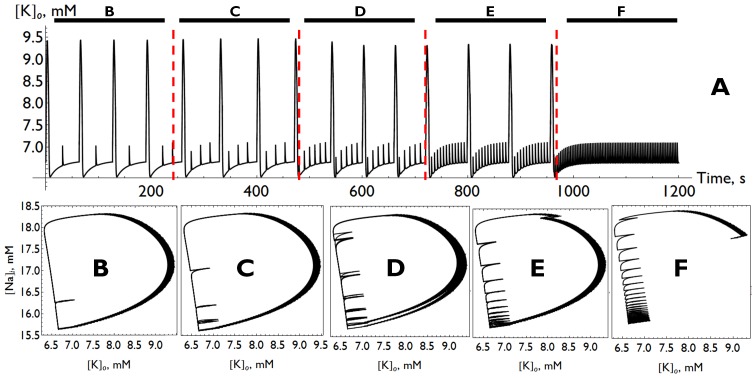
Stimulating at varying rates to trigger bursts: response skipping. At subthreshold 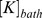 (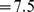 mM) it is possible to elicit bursts using large current injections. In this figure, all stimuli are 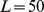 ms, 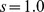 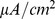. The model neuron cannot burst more quickly than a certain intrinsic frequency, even if the stimulation is applied more frequently. Panel (**A**) shows the potassium ion concentration over 20 minutes as the stimulation frequency is increased. Every 4 minutes the frequency is increased by a factor of 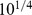, starting at 0.0316 Hz when 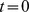. The evolution of the system at each individual frequency is shown in phase planes (**B–F**), which correspond to the identically labeled epochs of the voltage time trace above.

To conclude, we return to very low-frequency stimulation (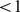 Hz), and examine the phenomenon of response skipping. Figure 7 shows the response of the model to successive four-minute epochs of stimulation in which the frequency increases in steps for each stimulation epoch. We set 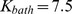 mM (i.e., no spontaneous bursting). Panel (A) shows 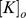 versus time. In the first epoch, labeled B, the stimulation consists of one stimulation pulse every 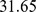 seconds (approximately), and a large response is observed on every other stimulation. Panel (B) shows this behavior in the ion concentration space. The large loop is the large transient response, and the short horizontal line segment emanating from the left edge of the loop is the attenuated response. Here, a large response did not occur because the ion concentrations had not yet recovered sufficiently from the previous burst. In particular, internal sodium remained high (and the external potassium was low) at the time the stimulation arrived, and it did not shift the system across the spiking and bursting thresholds described above. By the time the next stimulation arrives, however, the system has reached the lowest corner of the loop, and the stimulation does indeed evoke a large response.

In epochs C–F, stimulations arrive more frequently, and more small responses accumulate along the left edge of the ion concentration loops. This continues until, in panel (E), a stimulation deforms the upper part of the loop. In panel (F), the stimulation occurs sufficiently frequently so as to drive the system to the pseudoequilibrium in the lower-left corner, and no further large transient responses occur.

The simulations summarized in Figure 7 reveal that faster stimulation does not elicit more frequent burst-like responses. Instead, one sees “skipped responses”. Similar behavior has been observed in experimental seizure models [19].

## Discussion

In this paper, we have investigated the effects of periodic stimulation on a computational model of a single, Hodgkin-Huxley type neuron, extended to include the local intra- and extracellular ion concentration dynamics. Without stimulation, this model exhibits periodic bursting, which comes about as a result of the model’s intrinsic nonlinear dynamics. For appropriately-chosen parameter values, the ion concentrations oscillate periodically, driving the transmembrane voltage into and out of resting and spiking regimes. We have proposed this as a possible mechanism that may underlie seizures and/or seizure-like events in brain tissue.

We have shown that periodic stimulation interacts with and perturbs the ion concentration dynamics in such a way that bursting can be effectively controlled. The stimulation drives the system to steady states, which we call pseudoequilibria, in which the ion concentrations remain essentially constant, while the voltage either exhibits tonic spiking at the rate of stimulation (under excitatory stimulation), or quiescence (under inhibitory stimulation). We understand this behavior based on nonlinear dynamical analysis; the pseudoequilibria are actually very small limit cycles that occur in regions of the state space where the model’s intrinsic dynamics and the effects of stimulation balance each other out. We find that the stimulated neuron settles to this balanced state naturally for a wide range of parameters. That is, control is achieved without having to precisely tune the stimulation.

Further nonlinear dynamical analysis led to the notion of time-averaged “effective nullclines”. These are not true nullclines, which for our high-dimensional system would be (correspondingly) high-dimensional objects. However, because our system exhibits sufficient timescale separation, it is possible to identify curves in the space of ion concentrations where the *average* rate of change of 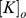 or 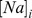 is zero. These effective nullclines are useful for describing the behavior of the slow ion concentration dynamics. In particular, we showed that the potassium nullcline acts as a threshold that clarifies the occurrence of a neuron’s short, attenuated response to an abrupt perturbation, or the occurrence of an explosive, seizure-like event, as illustrated in Figure 5.

Our model has several shortcomings. We do not model calcium, chloride, bicarbonate, pH, osmolarity, immobile anions, neuronal morphology, aerobic metabolism, or any but the most rudimentary ion channels and cotransporters. Furthermore, we have neglected the electrogenic contribution of the Na/K pump in the equation for the transmembrane voltage (although the inclusion of this term leaves the qualitative aspects of the model’s dynamical behavior and structure intact). Despite these abstractions, our model appears to successfully capture a portion of the gross dynamical behavior of real neuronal systems, suggests a minimal model that exhibits these interesting behaviors, and may have qualitative predictive and/or explanatory power.

Since the present work deals exclusively with a single neuron model, we neglect synaptic mechanisms. Extending our model to networks would require modeling transmitters including AMPA and GABA (and, therefore, the associated chloride dynamics). It would also vastly increase the parameter space to explore–for instance, there are many possible choices of network topology.

### Empirical Evidence

Despite the many obvious limitations of our model, there are a number of phenomena seen in experimental studies that might be at least partially understood in terms of the mechanisms that we have investigated here.

We showed that in our model, periodic stimulation stops bursting/seizing (Figure 1). This effect has been seen *in vitro* (slice preparation) and in cultured neurons, e.g. [19]–[22]. In these experimental studies, stimulation was found to disrupt pharmacologically- and genetically-induced seizures over particular frequency ranges. In the *in vitro* studies, seizures were successfully suppressed. In the cell culture work, even though stimulation did not completely halt seizure-like discharges at the frequencies investigated, it did significantly reduce their duration and frequency.

It may be that periodic stimulation is naturally employed in the brain as a control mechanism. There is evidence that interictal bursting in the CA3 region of the hippocampus exerts control on the CA1 region in this way. Work by Avoli et al. using *in vitro* models of epilepsy found that the frequency of these interictal bursts is anticorrelated with the occurrence of seizures [23]. Removing these intrinsic inputs by severing the Schaffer collaterals in mouse hippocampal-entorhinal slice preparations results in increased seizure activity in the entorhinal cortex in 4-aminopyridine and low magnesium models of epilepsy. Applying low frequency electrical stimulation (0.25–1.5 Hz) to the CA1 region, so as to mimic lost CA3 output (which previously consisted of interictal activity), restores control in the entorhinal cortex [24], [25]. These results closely parallel the mechanism we discuss, particularly insofar as it is possible to substitute synaptic signalling (along the Schaffer collaterals) with artificial stimulation, thus underlining the generality of the control mechanisms at work.

Tetanic stimulation is commonly used to generate seizure discharges experimentally. As in our model, the properties of the discharges elicited in this way depend on the stimulation parameters as well as the pre-stimulation state. For example, most *in vitro* models rely on some form of pharmacological manipulation or kindling in order to render the target cells susceptible to seizure generation by stimulation.

It has been observed that significant time, on the order of minutes, must elapse between stimulations in order for each to produce a maximal response. One study employing tetanic stimulation to evoke seizures in slice preparations found that seizure-inducing stimulations were optimally effective when they were applied at least ten minutes apart [26]. Stimulation at higher frequencies produced seizures of diminished strength. Work in cell culture has demonstrated reduction in seizure output with increasing stimulation frequency as well as with spontaneous inter-burst frequency [22]. As we have shown, similar behavior seen in our model can be explained by the model neuron’s location in the ion concentration space, in particular its sodium concentration, as it recovers from a sustained discharge. This result is consistent with [27], in which the role of sodium accumulation in burst termination and postictal depression was studied computationally.

In addition to a reduction in response strength, our model also predicts that the system’s location relative to the potassium nullcline determines whether a given input will produce a small or a sustained response. In our model, the trajectory of the system after recovery from a burst approaches this nullcline, suggesting that some stimulation protocols should give rise to alternating large and small responses. This phenomenon, seen in our model (see Figure 7), has been observed both in cell culture and *in vitro* [19], [22].

Gluckman et al. observed seizures in slice preparation immediately after a controlling, hyperpolarizing electric field was turned off [28]. They used a closed loop controller, i.e. simultaneous extracellular recordings were fed into an algorithm which adaptively controlled the applied electric field. A similar phenomenon is seen in our model–inhibitory stimulation sends the model to a low sodium region of the phase space where releasing control suddenly elicits a large burst.

Recent clinical studies have investigated the effectiveness of electrical stimulation for seizure control. This motivates the desire to better understand the mechanisms underlying this effect. For a number of recent reviews on therapeutic stimulation for epilepsy, see [29]–[32], and for an extensive list of clinical studies see [33]. Notable among recent studies is the large, double blind SANTE trial, which studied electrical stimulation of the anterior nuclei of the thalamus. Despite many reported side effects, the study observed seizure frequency reduction described as significant [3]. Some studies have seen effects at frequencies as low as or even lower than those our model predicts [4], [5], [34].

As we have mentioned before, our results do not require that control stimulation be supplied artificially. The control mechanism we describe is based on ionic dynamics–any kind of stimulation that perturbs the ion concentrations will have similar effects. The mechanism we describe, therefore, may also have bearing upon “remote” methods of controlling seizures, such as vagus nerve stimulation [35], which are currently thought to work by triggering inhibitory or excitatory neuronal pathways.

### Predictions

Several of our results have not been observed in experiment, as far as we know. These constitute testable predictions of our model.

In our model, we found that control achieved using periodic stimulation becomes more robust to increases in external potassium concentration with increasing frequency (Figure 3). In our model, this can be understood in terms of the locations of the SNIC curve and the potassium nullcline. It is, of course, important to remember that although high-frequency stimulation is more robust in this sense, it is potentially more invasive, for both energetic and information-related reasons. Higher spike rates lead to higher steady-state internal sodium concentrations that in turn cause pump rates and ATP consumption to increase. And each stimulation-induced spike will interfere with normal cellular signalling–an effect that only grows more disruptive with frequency. Physiologically, high frequency stimulation can lead to branch point conduction failure and synaptic plasticity effects [36]–[38].

We also showed that, in our model, qualitatively different transients occur in response to abrupt changes in 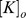 of different magnitudes (Figure 5). As above, this effect is explained in our model by reference to the locations of the various thresholds in the ion concentration phase space. Similar behavior in response to more realistic inputs can be understood in the same way (Figure 6). To the best of our knowledge, no experimental studies have directly investigated the precise relationships between seizure discharges, stimulation parameters, and the composition of the bath solution.

Finally, we found that seizure-like transients can occur when periodic stimulation is turned on, but that these can be mitigated by adjusting 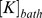 or the stimulation frequency slowly (Figure 4). When these parameters are increased slowly, on the order of seconds, the system can settle into a pseudoequilibrium without a large (potentially harmful) excursion in the phase space.

The careful experimental exploration of the stimulation parameter space to find optimal strategies for seizure control is a crucial prerequisite to deploying stimulation-based therapies. Unfortunately, this parameter space is very large [39], particularly if we consider that the optimality of a stimulation protocol may depend on the type of epilepsy being tackled or neuroanatomical structure being targeted. Nevertheless, mathematical models can be useful in guiding the search, as they can generate hypotheses to be refined in experiment [1].

### Conclusion

The trafficking of ions across the neuronal membrane–carefully orchestrated by voltage-gated ion channels and other transporters–gives rise to the electrophysiological characteristics of the neuron on which its function crucially depends. Unsurprisingly therefore, the failure of systems which control a neuron’s ionic environment have been strongly implicated in seizures and epilepsies.

We computationally analyzed a simple mathematical neuron model which exhibits seizure-like oscillations driven by abnormal ion concentration dynamics. Periodically perturbing the model, as if to mimic electrical stimulation of the neuron, can stop these oscillations. It is also possible to use stimulation to make the model neuron less excitable.

Electrical stimulation is well established as a treatment for movement disorders, and now, backed by many studies, is gaining momentum as a potential therapy for individuals with otherwise intractable epilepsies. However, the large space of possible stimulation parameters and lack of consensus regarding the mechanism of action make rational therapy design challenging. We believe our results, which place therapeutic stimulation for epilepsy in the context of ion concentrations, add an important layer to the story.
